# Psychological feelings and behavioral changes in patients with fear of insulin injection and blood glucose monitoring: A qualitative study in China

**DOI:** 10.1097/MD.0000000000049570

**Published:** 2026-07-03

**Authors:** Juan Zhang, Xueyu Zhang, Jie Zhou, Jing Yang, Adan Fu

**Affiliations:** a Department of Endocrinology, The Central Hospital of Wuhan, Tongji Medical College, Huazhong University of Science and Technology, Wuhan, Hubei, China; b Department of Nursing, The Central Hospital of Wuhan, Tongji Medical College, Huazhong University of Science and Technology, Wuhan, Hubei, China.

**Keywords:** behavioral characteristics, blood glucose monitoring, diabetes, fear, insulin therapy, psychological feelings, qualitative study

## Abstract

The fear associated with insulin injections and blood glucose monitoring is often overlooked in clinical practice. Although patients are fully aware of the importance of insulin injections and blood glucose monitoring, fear can significantly reduce their treatment compliance, and the resulting psychological and behavioral changes may further exacerbate this negative impact. Therefore, it is imperative to employ qualitative research to thoroughly explore the patterns of patients’ psychological feelings and behavioral changes. This study aimed to investigate the characteristics of psychological feelings and behavioral changes in patients with a fear of insulin injections and blood glucose monitoring, thereby providing a scientific basis for improving their self-management abilities. This study adopted a phenomenological approach within the framework of qualitative research. The participants were patients with a fear of insulin injections and blood glucose monitoring. Data were collected through semi-structured, face-to-face, in-depth interviews and analyzed using Colaizzi’s 7-step analysis method combined with microanalysis. Four themes emerged that reflected the psychological feelings and behavioral manifestations of patients with a fear of insulin injections and blood glucose monitoring: “worry–fear and behavioral imbalance,” “denial–resistance and activation of defense mechanisms,” “anxiety–irritability and depletion of self-efficacy,” and “compromise–acceptance and establishment of adaptive behaviors.” The emotional states of patients with a fear of insulin injections and blood glucose monitoring exhibited regular phase changes, each accompanied by corresponding behavioral characteristics. Therefore, in clinical practice, medical educators should develop differentiated intervention and correction strategies based on the emotional and behavioral characteristics of patients in different phases.

## 1. Introduction

By 2023, the global population of people with diabetes aged 20 to 79 years had reached 537 million, with projections indicating an increase to 783 million by 2045.^[1]^ Diabetes is a globally prevalent chronic metabolic disorder that poses a major public health challenge. Insulin therapy is crucial for controlling blood glucose levels and reducing the risk of complications, and it requires lifelong management and treatment through individual lifestyle changes and adjustments.^[2]^ Self-management is a key determining factor in diabetes management; however, certain fears and anxieties can hinder disease self-management.^[3]^ Previous studies have indicated that patients with diabetes receiving insulin therapy commonly experience a fear of insulin injections and blood glucose monitoring. This phenomenon can trigger psychological insulin resistance and delay the initiation of insulin treatment, leading to negative self-management behaviors that accelerate the onset of complications and increase the risk of mortality.^[4]^ Therefore, enhancing patients’ self-management abilities is essential for preventing both short- and long-term acute and chronic complications. Currently, existing relevant studies in China mostly focus on fear scale compilation as well as intervention strategy discussions and practices.^[5–7]^ However, no research has explored these fears based on patients’ subjective feelings and real experiences. In this context, this study used phenomenological research methods to conduct semi-structured, in-depth interviews with patients experiencing a fear of insulin injections and blood glucose monitoring. This study aimed to elucidate the psychological feelings and common behavioral characteristics associated with this fear and to develop effective behavioral intervention strategies for fear prevention, with the ultimate goal of enhancing treatment efficacy and improving quality of life.

## 2. Methods

### 2.1. Design

This study adopted a phenomenological research approach and adhered to the Standards for Reporting Qualitative Research guidelines. Colaizzi’s 7-step analysis method^[8]^ and the microanalysis technique^[9]^ were employed to explain and classify textual data.

### 2.2. Participants

Purposive sampling was used to recruit participants with diabetes who attended the Department of Endocrinology at the Central Hospital of Wuhan, Wuhan City, Hubei Province. Recruitment was conducted by the first author, who holds a Master of Nursing degree, and was supported by a diabetes specialist. The inclusion criteria were as follows: meeting the Chinese diagnostic criteria for type 2 diabetes^[10]^; age ≥18 years with a disease duration ≥6 months; receiving insulin injections and blood glucose monitoring for ≥6 months; a total score ≥6 on the Chinese version of the Diabetes Fear of Insulin Injection and Blood Glucose Monitoring Questionnaire^[11]^; good language expression and communication skills; and provision of informed consent and voluntary participation in the study. The exclusion criteria were as follows: inability to cooperate with the investigation because of comorbid diseases of other systems or cognitive impairment. Sampling followed the principle of maximum variation based on age, gender, and educational level. Sample size was determined using the data saturation principle, that is, when the information mentioned by the interviewees was similar to or repeated that of a previous participant.^[12]^ When interview data collection reached 15 cases, no new information emerged. Following the 16th participant interview, the data fully confirmed the saturation criteria. Thus, 16 patients with diabetes were included in this study. The interview participants were sequentially coded as P1 to P16.

### 2.3. Data collection

Data were collected between October 2022 and March 2023. All patients were informed of the study’s aim and agreed to participate in in-depth interviews. The interviews were one-on-one, face-to-face, semi-structured, and were conducted in a quiet, disturbance-free place where participants felt at ease, such as the ward’s health education room in the hospital. Each interview lasted 30 to 50 minutes. During each interview, the interviewer avoided using any leading or suggestive language, listened patiently, encouraged participants to express their inner experiences, and recorded their behavioral and emotional changes in real time. When the interview data became saturated and no new themes emerged, the interviews were concluded.

Based on the research objectives, an interview guideline was developed through a comprehensive literature review. Three participants who met the inclusion criteria were selected for pre-interviews. The final interview guideline was developed based on the results of the pre-interviews and group discussions (Table 1).

**Table 1:** Interview guideline.

1. What is your understanding of the fear associated with insulin injection and blood glucose monitoring?
2. What physical and psychological responses do you experience when this fear occurs?
3. In what ways has the fear of insulin injection and blood glucose monitoring impacted your life?
4. What strategies do you employ to manage and mitigate this fear?
5. What forms of assistance do you anticipate would help in overcoming this fear?

### 2.4. Data analysis

The recorded interviews were analyzed independently by 2 researchers: one listened to and transcribed the audio recordings into text within 24 hours of the interviews, and the other checked the transcriptions and returned them to the interviewees for confirmation. All respondents indicated that the transcriptions were correct, without modifications or additions. Subsequently, the research team discussed and extracted key statements to develop coded themes. In addition, a qualified expert in qualitative research was invited weekly to review the preliminary coding results and analytical logic, identify interpretive biases, propose revisions, and refine the final thematic framework.

### 2.5. Rigor

To ensure the trustworthiness of this study, all interviewers were certified diabetes specialist nurses who received standardized pretraining in phenomenological qualitative research, covering core qualitative methodologies and interviewing skills. Prior to participant recruitment, each interviewer reflected on and documented their preexisting assumptions regarding patients’ fear of insulin injections to identify potential subjective biases. Interviewers maintained ongoing reflexivity by writing regular research journals throughout the study. All face-to-face interviews were conducted strictly in accordance with the finalized semi-structured interview guide, with suggestive or leading questions avoided entirely. After each interview, interviewers completed reflexive logs to record personal subjective biases and emotional interferences that might compromise data authenticity. The research team held regular group meetings to review interview procedures and rectify inappropriate practices. Furthermore, the entire research process was supervised and guided throughout by a senior professor with extensive experience in qualitative research and academic teaching to guarantee methodological rigor.

### 2.6. Ethical considerations

This study was approved by the Ethics Committee of the Wuhan Central Hospital (approval No. WHZXKYL2022-210). Before the formal interview began, the researcher introduced herself and explained the purpose and content of the study. Participants were informed that the interview data would be used solely for this study and that their personal privacy would be strictly protected. Informed consent was obtained from all participants, and the interviews were audio-recorded. All participants voluntarily took part in this study, and none refused to participate or withdrew.

## 3. Results

### 3.1. General information of the interviewees

A total of 16 interviewees participated in this study. The average age was 47 ± 16.17 years. Among the participants, 5 (31.2%) were male and 11 (68.8%) were female; education level: 4 (25.0%) had an associate degree, 8 (50.0%) had a bachelor’s degree, 2 (12.5%) had a junior high school education, 1 (6.2%) had a secondary vocational school education, and 1 (6.2%) had a senior high school education; marital status: 13 (81.2%) were married; and 3 (18.7%) were unmarried (Table 2).

**Table 2:** Characteristics of participants.

ID	Sex	Age	Educational level	Marital status	Career	Medical expenses	Residential status	Time to diagnosis (yr)	Time for insulin injection (yr)	D-FISQ score
P1	Female	34	Bachelor’s degree	Married	Staff	Medical insurance	Living with family	1	1	15
P2	Female	76	Associate degree	Married	Retired	Medical insurance	Living with family	16	6	30
P3	Female	62	Bachelor’s degree	Married	Retired	Medical insurance	Living with family	19	5	57
P4	Female	42	Bachelor’s degree	Married	No occupation	Medical insurance	Living with family	1	1	17
P5	Male	39	Associate degree	Married	Worker	Medical insurance	Living with family	2.5	1.5	49
P6	Female	57	Junior high school	Married	Retired	Medical insurance	Living with family	10	10	35
P7	Male	33	Secondary vocational school	Married	Staff	Medical insurance	Living with family	9	9	19
P8	Female	66	Senior high school	Married	Retired	Medical insurance	Living with family	12	7	23
P9	Male	52	Bachelor’s degree	Married	No occupation	Self-payment	Living with family	5	2	11
P10	Female	20	Bachelor’s degree	Unmarried	Student	Medical insurance	Living with family	1.5	1	10
P11	Female	42	Bachelor’s degree	Married	Teacher	Medical insurance	Living with family	2	1	11
P12	Female	30	Associate degree	Unmarried	No occupation	Self-payment	Living alone	2	1	49
P13	Male	29	Bachelor’s degree	Unmarried	Civil servant	Medical insurance	Living alone	2	1	66
P14	Female	39	Bachelor’s degree	Married	Technical staff	Medical insurance	Living with family	4	3	11
P15	Female	71	Junior high school	Married	Retired	Medical insurance	Living with family	28	24	13
P16	Male	60	Associate degree	Married	Retired	Medical insurance	Living with family	8	8	15

D-FISQ = Diabetes Fear of Insulin Injection and Blood Glucose Monitoring Questionnaire.

### 3.2. Themes

This study distilled 4 themes: worry–fear and behavioral imbalance, denial–resistance and activation of defense mechanisms, anxiety–irritability and depletion of self-efficacy, and compromise–acceptance and establishment of adaptive behaviors. Emotional evolution exhibited phased changes accompanied by typical physical behavioral manifestations.

#### 3.2.1. Theme 1: worry–fear and behavioral imbalance

During treatment, patients frequently experience worry and fear associated with insulin injections and blood glucose monitoring. They are also concerned about the safety of the injection needles, such as insufficient disinfection and needle bending, as well as apprehensions about long-term medication side effects, including skin induration, hypoglycemia, and drug dependency. These fears and worries lead to cognitive dissonance regarding disease treatment, affecting patients’ treatment adherence and psychological state, thereby hampering effective disease management.

“Because diabetes makes me prone to infections and slow wound healing, I worry that the manufacturers didn’t properly disinfect the needles, and I also fear I won’t disinfect them well at home. Several times, the needles bent; they did. When I was injected, the medication would not push through, and when I pulled the needle out, I saw it bend. My hands often shake and my heart races when injecting; sometimes I sweat, and the injection site also feels stiff.”

(Participant 3)

“I’ve heard from others that excessive insulin injections can cause hard lumps under the skin, which I’m quite worried about.”

(Participant 7)

“I don’t know if long-term or lifelong insulin injections have side effects, or if I’ll become dependent on the drug. I think that too many injections might be bad for my health.”

(Participant 10)

“When it’s almost time for my insulin injection, I feel a bit nervous – restless, pacing back and forth. As soon as I pinched the skin on my abdomen, my hands started shaking.”

(Participant 12)

“When I first started monitoring my blood glucose, my hands were stiff, my muscles tense, and I could not bring myself to prick my finger.”

(Participant 15)

#### 3.2.2. Theme 2: denial–resistance and activation of defense mechanisms

Patients with diabetes experience psychological distress because of fear of injections and lifestyle changes. Long-term, frequent insulin injections further exacerbate patients’ avoidance behavior, causing them to feel rejection and anxiety about insulin injections and resistance to disease treatment. These emotional responses represent the activation of patients’ psychological defense mechanisms, which influence their acceptance of and adherence to treatment.

“I used to love eating snacks, but now I’m suddenly told I can’t. It makes me feel really bad – I still crave snacks so much, and I just cannot help but feel this sense of loss.”

(Participant 2)

“I’ve always been scared of injections, and now I have to take an insulin shot every day, plus prick my finger for blood glucose tests every day. I cannot accept this. Since I got this disease, the daily insulin injections felt terrifying to me, and I resisted it a lot.”

(Participant 4)

“I have to take insulin quite frequently – you see, four times a day. After a while, I started to reject it. I just wanted to avoid it, refused to acknowledge my illness, and did not want to inject anymore. In fact, I used to avoid monitoring my blood glucose. My family kept pushing me to do so, and we had arguments about it, which greatly affected me. When I see my classmates and colleagues – people of my age – who are healthy and can eat whatever they want, I feel envious. Over time, I began to feel slightly inferior.”

(Participant 5)

#### 3.2.3. Theme 3: anxiety–irritability and depletion of self-efficacy

Patients with diabetes are prone to persistent anxiety and psychological burden owing to fear of daily injections and concern about elevated blood glucose levels. This long-term anxiety and psychological pressure further aggravate patients’ self-efficacy and affect their quality of life and disease management ability.

“I take insulin once every morning, and it is like a conditioned reflex – I know I am going to feel pain at this time every day, and this anxiety is always with me.”

(Participant 1)

“After I started taking insulin, my temper became very short. I get angry easily when helping my child with homework at home; nothing seems right to me, and I do not want to talk about my illness at all.”

(Participant 4)

“Every time I monitor my blood glucose, I have to wait for five seconds, and I feel quite anxious – afraid that the result will show elevated blood glucose, which might lead to complications later.”

(Participant 6)

“I used to be very outgoing, but now I’m depressed. I do not want to communicate much with others; I really just want to shut myself off. Sometimes I feel very irritable and easily lose my temper.”

(Participant 9)

“I’ve been afraid of pain since I was a child, and I’m also quite scared of injections. So, I feel restless when I have to take insulin before meals.”

(Participant 12)

“Since I got this disease, I can’t stay up late or go out with friends anymore. I feel disconnected from society.”

(Participant 13)

#### 3.2.4. Theme 4: compromise–acceptance and establishment of adaptive behaviors

To maintain health, patients with diabetes must adhere to treatment regimens, including injections, when managing their condition. Moving from initial resistance to helpless compromise and eventual acceptance, they gradually established adaptive behaviors, such as self-adjustment, distraction, and emotional relief, to maintain their life beliefs and health goals. This psychological transformation reflected their hopes for the future and their pursuit of health.

“I have to endure it to save my life. After all, I need to rush to work immediately after the morning injection.”

(Participant 7)

“I’ve always been afraid of pain, but I have no choice – I have to take the injection. I need to be responsible for my health, so my condition can be well controlled.”

(Participant 8)

“I’ll ask my family to hold my finger tightly, then prick it quickly. If I really cannot bring myself to do it, I will go to the hospital outpatient department to check my blood glucose level.”

(Participant 11)

“I’ve always been scared of injections, but there’s no other way. To keep living and stay healthy, I still believe in science – and with treatment, my condition will get better.”

(Participant 13)

“I’ll sit down and calm myself for a minute or two. Then, I will play some really upbeat or fast-tempo music, and I will also massage the area a bit to relax myself when I am nervous.”

(Participant 14)

## 4. Discussion

Emotional experience refers to an individual’s psychological responses and feelings toward illness, including personal perceptions and experiences regarding disease progression, treatment procedures, prognosis, and life changes. Positive emotions benefit disease treatment, rehabilitation, and quality of life across multiple dimensions.^[13]^ This study found that patients with diabetes undergoing insulin therapy commonly experience dynamic and complex emotional changes, shifting from initial worry, fear, denial, anxiety, and irritability to gradual acceptance and behavioral compromise. Consistent with previous evidence,^[14]^ such treatment-related negative emotions among patients with diabetes are not merely passive byproducts of treatment but rather crucial intrinsic factors that undermine treatment adherence. These affective responses directly trigger avoidant behaviors, such as delayed insulin injections and reduced blood glucose monitoring, and may further develop into long-term maladaptive coping strategies. Patient emotions are dynamically shaped by physiological fluctuations, life events, and prior negative disease experiences.^[15]^ Only when emotional experience is regarded as a core treatment element and dynamically integrated throughout the entire process can the deep motivation for behavioral change truly be activated. Therefore, in-depth identification and targeted guidance of patients’ emotional experiences are essential to facilitate positive behavioral modification. Healthcare providers should offer stage-specific, tailored interventions based on patients’ emotional trajectories: for patients in the initial fear phase, live demonstration with step-by-step instruction is recommended; for those in the denial or anxiety phase, a guided, encouraging approach should be used to help patients establish cognitive reframing, combined with diaphragmatic breathing relaxation techniques to alleviate anxiety; and once patients reach the acceptance phase, online and offline case-based education should be employed to help consolidate their acceptance behaviors.

Disease cognition refers to patients’ comprehensive understanding, beliefs, and emotional perceptions of their illness, which profoundly determines treatment adherence and self-managementbehaviors.^[16]^ Accurate and scientific disease cognition promotes treatment adherence, whereas insufficient or distorted cognition exacerbates disease-related fear, induces cognitive dissonance, and impedes effective disease management. In this study, most participants exhibited cognitive misunderstandings regarding insulin therapy. Excessive concerns about needle safety and overattention to the potential long-term side effects of repeated injections led to negative psychological states and poor treatment outcomes. These findings are consistent with previous studies focusing on cognitive deficits in patients with diabetes, including insufficient disease knowledge, inadequate awareness of blood glucose monitoring, and limited understanding of chronic complications.^[17]^ Empirical evidence further confirms that patients’ level of disease cognition is positively correlated with medication adherence and self-management ability, highlighting the necessity of correcting cognitive biases to optimize diabetes management.^[18]^ Existing studies also emphasize that health education should be individualized according to patients’ cognitive characteristics, with the improvement of disease cognition regarded as a core intervention goal for standardizing health behaviors and enhancing adherence.^[19]^ In summary, systematic and continuous health education is crucial for patients with diabetes. Through ongoing health management education,^[20]^ healthcare providers can use anatomical diagrams to illustrate the depth of subcutaneous injection and compare injection site safety with the risks of chronic hyperglycemia, thereby guiding patients to develop a rational understanding of the necessity and safety of insulin therapy and eliminating barriers caused by misconceptions.

Diabetes management is a comprehensive process that integrates physiological, psychological, and social dimensions. During long-term treatment, patients become trapped in the dual low-efficiencydilemma of persistent social pressure and an internal psychological gap, which ultimately impairs their disease management abilities and quality of life. Our study found that insulin therapy not only affects patients themselves but also profoundly impacts their social roles and daily lives. Consistent with the findings of Jiang et al,^[21]^ our analysis indicates that patients require dietary and lifestyle adjustments because of insulin injections and often face misunderstandings from others, which frequently limit their social activities and may even lead to social avoidance behaviors. Azmiardi et al^[22]^ pointed out that low social support is a significant risk factor for diabetes complicated by depression, whereas a strong social support network can effectively buffer stress and maintain physical and mental health.^[23]^ Therefore, establishing and activating a multidimensional support network that encompasses hospitals, communities, families, and peers has become a core pathway to enhancing patients’ self-management abilities. In clinical practice, healthcare providers should build upon the stage-specific interventions described above and adopt tailored strategies based on patients’ behavioral presentations. For example, for patients with prominent fear-avoidance behaviors, graded exposure therapy can be used, progressing stepwise from watching a demonstration, to handling the device, to simulated injection, and finally to actual injection. For patients who are prone to giving up or procrastinating, small, achievable goals should be set; upon goal completion, immediate positive feedback should be provided along with validation of their feelings, and the difficulty of goals should be gradually increased. In addition, the modeling effect of peer support^[24]^ can be leveraged to enhance patients’ self‑management abilities and achieve sustainable disease management.

## 5. Conclusion

In this study, we conducted in-depth interviews with 16 patients experiencing fear of insulin injections and blood glucose monitoring and found a regular evolution of emotion during insulin therapy and blood glucose monitoring, accompanied by common behavioral characteristics, including worry–fear and behavioral imbalance, denial–resistance and activation of defense mechanisms, anxiety–irritability and depletion of self-efficacy, and compromise–acceptance and establishment of adaptive behaviors. Therefore, healthcare providers should be encouraged to place emotional assessment at the core of insulin therapy. They should dynamically monitor emotional changes in patients with diabetes who fear insulin injections and blood glucose monitoring and provide timely emotional support based on the unique emotional characteristics present at each stage. Additionally, a systematic and continuous care education model must be implemented for patients with diabetes to dismantle misconceptions, reconstruct their disease knowledge framework, and activate a multidimensional support network. This approach enhances patients’ self-efficacy and collectively drives their transformation from passive recipients of treatment to active health advocates.

## 6. Limitations

This study was a single-center investigation with a sample drawn only from Hubei Province, China, and the sample size was limited. Future research should expand the sample size and include populations from different countries and regions to enable cross-cultural comparisons. Furthermore, the findings may be influenced by the China-specific cultural background, and the shaping effect on injection fear may differ from that in other cultural contexts. Additionally, this study was conducted within a tertiary hospital healthcare system in China, where the allocation of support resources and patient–provider interaction patterns may differ from those in other countries.

Despite the above geographical limitations, this study has unique strengths. It provides an in-depth exploration of the culturally specific perceptions and intrinsic characteristics of insulin injection fear among patients with diabetes in the Chinese healthcare context, helping to elucidate the potential influence of cultural factors on patients’ fear experiences. Moreover, the results offer localized qualitative evidence for future cross-cultural comparisons and lay a foundation for further validating the transferability of the relevant conclusions to healthcare settings outside China. Future multicenter, large-sample, cross-regional studies are necessary to verify and extend the applicability of the current findings to broader populations.

## Acknowledgments

We thank the authors of all the original papers included in this study, as well as the participants. We also appreciate the editorial office and reviewers for their careful evaluation, insightful comments, and hard work.

## Author contributions

**Conceptualization:** Juan Zhang, Adan Fu.

**Funding acquisition:** Juan Zhang.

**Methodology:** Juan Zhang.

**Project administration:** Juan Zhang.

**Data curation:** Xueyu Zhang, Jie Zhou.

**Supervision:** Jing Yang, Adan Fu.

**Writing – original draft:** Juan Zhang, Xueyu Zhang.

**Writing – review & editing:** Jing Yang.

## References

[1] Sun H Saeedi P Karuranga S. IDF Diabetes Atlas: global, regional and country-level diabetes prevalence estimates for 2021 and projections for 2045. Diabetes Res Clin Pract. 2022;183:109119. 34879977 10.1016/j.diabres.2021.109119 PMC11057359

[2] Xie M Chen S He Q. Survey of fear and compliance of insulin degludec and insulin aspart injection in type 2 diabetes mellitus patients and analysis of influencing factors. Medicine (Baltimore). 2024;103:e40286. 39533598 10.1097/MD.0000000000040286 PMC11556997

[3] Töyer Şahin N Ek H Pehlivan S. The relationship between discomfort intolerance and the fear of self-injection and testing in patients with diabetes using insulin: a cross-sectional study. J Clin Nurs. 2025;34:2840–51. 39370576 10.1111/jocn.17482 PMC12181152

[4] Celik S Pinar R. Psychometric evaluation of a Turkish version of the Diabetes Fear of Self-Injecting and Self-Testing Questionnaire (D-FISQ). Asian Nurs Res (Korean Soc Nurs Sci). 2016;10:195–200. 27692248 10.1016/j.anr.2016.06.001

[5] Snoek FJ Mollema ED Heine RJ Bouter LM van der Ploeg HM. Development and validation of the Diabetes Fear of Injecting and Self-Testing Questionnaire (D-FISQ): first findings. Diabet Med. 1997;14:871–6. 9371481 10.1002/(SICI)1096-9136(199710)14:10<871::AID-DIA457>3.0.CO;2-Y

[6] Mollema ED Snoek FJ Pouwer F Heine RJ van der Ploeg HM. Diabetes Fear of Injecting and Self-Testing Questionnaire: a psychometric evaluation. Diabetes Care. 2000;23:765–9. 10840993 10.2337/diacare.23.6.765

[7] Jin W Liu HH Fu YL. Effect of nurse-led stress inoculation intervention on fear of insulin injection and blood glucose monitoring in elderly patients with type 2 diabetes mellitus. Chin J Pract Nurs. 2022;38:513–8.

[8] Liu M. Application of Colaizzi’s seven-step method in data analysis of phenomenological research. J Nurs Sci. 2019;34:90–2.

[9] Chen XM. Qualitative Research Methods and Social Science Research. Educational Science Publishing House; 2002.

[10] Chinese Diabetes Society. Guidelines for the prevention and treatment of type 2 diabetes in China (2020 edition). Chin J Diabetes Mellitus. 2021;13:482–548.

[11] Liu XQ. Current Status and Intervention Study on Fear of Insulin Injection and Self-Monitoring in Children with Type 1 Diabetes [dissertation]. Chongqing: Chongqing Medical University; 2023.

[12] Song C Yao L. The experience of social alienation in elderly lung cancer patients: a qualitative study. Asian Nurs Res (Korean Soc Nurs Sci). 2024;18:281–7. 39089442 10.1016/j.anr.2024.07.007

[13] Rodríguez-Fuertes A Reinares-Lara P Garcia-Henche B. Incorporation of the emotional indicators of the patient journey into healthcare organization management. Health Expect. 2023;26:297–306. 36335577 10.1111/hex.13656 PMC9854301

[14] Resurrección DM Navas-Campaña D Gutiérrez-Colosía MR Ibáñez-Alfonso JA Ruiz-Aranda D. Psychotherapeutic interventions to improve psychological adjustment in type 1 diabetes: a systematic review. Int J Environ Res Public Health. 2021;18:10940. 34682687 10.3390/ijerph182010940 PMC8535719

[15] Liu HL. Application of Health Education Based on the IMB Model in Diabetes-Related Distress among Patients with Type 2 Diabetes Mellitus. Kunming Medical University; 2024.

[16] Xiong C He Y Zhang Y. Relationship between illness perception and self-management behaviors among Chinese diabetic foot patients. Jpn J Nurs Sci. 2023;20:e12550. 37477049 10.1111/jjns.12550

[17] Chen J Zhou JY Ji XJ. Intervention effect of MMC targeted management strategy on disease cognition, quality of life and glycemic control in patients with type 2 diabetes mellitus. Int J Nurs. 2025;44:612–6.

[18] Eshete A Getye B Aynaddis G. Association between illness perception and medication adherence in patients with diabetes mellitus in North Shoa, Zone: cross-sectional study. Front Public Health. 2023;11:1214725. 38174073 10.3389/fpubh.2023.1214725 PMC10762864

[19] Bilondi SS Noghabi AD Aalami H. The relationship between illness perception and medication adherence in patients with diabetes mellitus type II: illness perception and medication adherence. J Prev Med Hyg. 2021;62:E966–71. 35603233 10.15167/2421-4248/jpmh2021.62.4.2277 PMC9104663

[20] Wang Y Xue J Hu J. Study on the effect of dynamic health education on anxiety, depression and self-efficacy in pre-elderly patients with gastric cancer. Cancer Progression. 2018;16:123–5, 127.

[21] Jiang T Wang YL Zhang Y. Correlation study on negative emotions, coping strategies and psychological resilience in hospitalized young patients with type 2 diabetes mellitus. J Anhui Med Univ. 2025;60:524–35.

[22] Azmiardi A Murti B Febrinasari RP Tamtomo DG. Low social support and risk for depression in people with type 2 diabetes mellitus: a systematic review and meta-analysis. J Prev Med Public Health. 2022;55:37–48. 35135047 10.3961/jpmph.21.490 PMC8841196

[23] Ramkisson S Pillay BJ Sibanda W. Social support and coping in adults with type 2 diabetes. Afr J Prim Health Care Fam Med. 2017;9:e1–8. 10.4102/phcfm.v9i1.1405 PMC5566130 28828879

[24] Wang J Zou Y Liu RH. Path analysis of the effects of self-efficacy, illness perception, and social support on psychological resilience in patients with type 2 diabetes mellitus. Nurs Res. 2018;32:3729–32.

